# Treatment of Malignant Œdema with Carbolic Acid

**Published:** 1902-05

**Authors:** Charles F. Dawson

**Affiliations:** Lake City, Fla.


### TREATMENT OF MALIGNANT ŒDEMA WITH CARBOLIC ACID, WITH REMARKS UPON "LEECHES" OR BURSATTEE.

By Charles F. Dawson, M.D., D.V.S.,
LAKE CITY, FLA.

The patient, a native three-year-old pony, was brought to me, suffering with a "leech" tumor, which involved the tissues, including bone, from hock to fetlock. The animal was cast and kept under chloroform for two hours by green students. During the operation of removal of the tumor we found it advisable to administer a stimulant, and dilute alcohol was injected into the costal region near the point of the elbow. The next day considerable tenderness was shown at the site of the injections. The next day a large crepitating tumor had formed. A diagnosis of malignant œdema was made.

We considered it highly important to save the animal's life in order to make a test of the curability of a "leech" tumor of six-months' growth. Therefore, any method which promised a cure of the malignant œdema was eagerly seized upon. I decided to inject strong carbolic acid into the tumor in one-drachm doses on three succeeding days. There was complete subsidence of the gas tumor. The animal's condition was pretty low, but I noticed no particular symptom which could be attributed to the carbolic acid. There was rapid death of the subcutaneous connective tissue for the space of twelve inches square, and the problem now confronting us was to get rid of the dead tissue. An operation for its mechanical removal was planned and abandoned. Poultices were applied instead, and in a day or two I was gratified to find liquefaction in progress.

The abscess, which pointed, was lanced, thus liberating considerable pus and allowing the mechanical removal of several large pieces of dead tissue. A seton-needle threaded with a drainage-tube was entered at the opening and passed downward to the lowest margin of the dead area and out through the skin. Liquefaction was aided by daily baths of warm water, and the wound was irrigated with 1 per cent. creolin. A tonic of quinine, iron, nux vomica, and alcohol was given regularly.

Although the animal seemed on many occasions to be dying, it made a good recovery in a month, and was returned to the owner. Barring a slight luxation of the fetlock-joint, from weakening of the extensor pedis tendon from the "leech" disease, the animal walked normally, although the leg had not skinned over, nor could a favorable prognosis as to the non-recurrence of the "leech" tumor be given.

As some practitioners may not have seen this disease, it may be well to state that "leeches" or bursattee is a common disease in Florida, which manifests itself in the formation of tumor-growths which have some of the characters of the actinomycotic tumors. Its structure is fibrous, and contains many sinuses, which discharge a bloody, honey-like fluid. It is a fatal, infectious disease, which has its origin in the skin, and finally penetrates all the tissues. Here and there in the tumor tissue yellow bodies with root-like projections may be found. These bodies are called "leeches" by the natives. They consist of the mycelia of the fungus which causes the disease. The only remedy is complete removal of the tumor and adjacent tissue at once. The application of caustics and disinfectants makes matters infinitely worse, as they stimulate the tissues to renewed growth-activity. In Florida the disease affects only the genus equus.

